# Case Report: Extreme resistance to subcutaneous insulin in a cirrhotic patient with new-onset diabetes that resolved after transplantation

**DOI:** 10.3389/fendo.2025.1464532

**Published:** 2025-05-26

**Authors:** Kim Tunez, Sara Ringwald-de Meyer, Mohammed Barigou, Christophe Kosinski

**Affiliations:** ^1^ Service of Endocrinology, Diabetes and Metabolism, Department of Medicine, Lausanne University Hospital (CHUV) and University of Lausanne, Lausanne, Switzerland; ^2^ Service of Endocrinology and Diabetes, Geneva University Hospitals (HUG), Genève, Switzerland

**Keywords:** cirrhosis, diabetes mellitus, new onset diabetes, liver transplantation, subcutaneous insulin resistance

## Abstract

Although an increase in insulin resistance is frequently associated with liver cirrhosis, a condition sometimes referred to as hepatogenic diabetes (HD), the pathophysiology is still poorly understood. Moreover, the management of patients with HD, both diagnostically and therapeutically, is complex. In this article, we present the case of a cirrhotic patient, initially not known to have diabetes or prediabetes, who developed fulminant diabetes overnight, with massive resistance to subcutaneous insulin treatment. Management during hospitalization was extremely complicated, leading to significant staggering of the insulin doses until the liver transplant, which allowed complete normalization of the glycemic profile.

## Introduction

Individuals suffering from liver cirrhosis frequently experience increased insulin resistance (IR) by reduced insulin sensitivity and impaired glucose disposal due to defective glucose storage (1). It could be hypothesized that the mechanism is primarily driven by liver damage and a decreased expression of the insulin receptors in peripheral tissues. The risk factors, diagnosis, and treatment differ significantly from those of type 2 diabetes mellitus (T2DM) (2). The pathophysiology, which appears directly related to parenchymal cell damage, portosystemic shunt, and the liver disease etiology, remains poorly understood, as do the treatment options (3, 4). Herein, we report a case of extreme IR that developed in a short period of time, necessitating significant insulin requirements.

## Case description

A 51-year-old Caucasian woman, with a known history of Child-Pugh stage B9 cirrhosis and a Model for End-Stage Liver Disease (MELD) score of 34, resulting from a mixed etiology of alcohol use and chronic hepatitis B infection diagnosed in 2016, was listed for liver transplantation. She was hospitalized in October 2022 at Lausanne University Hospital (CHUV), Switzerland, for a decompensated cirrhosis with refractory chylous ascites. Her condition was complicated by hepatorenal syndrome and spontaneous bacterial peritonitis with hepatic encephalopathy, necessitating multiple intensive care unit stays.

**Figure 1:**
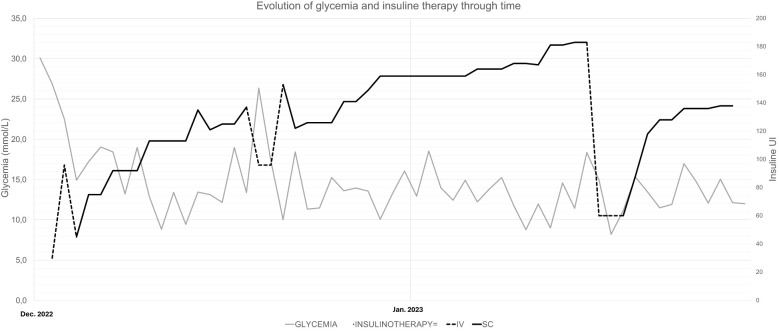
Evolution of glycemia and insulin through time.

The patient had no previous history of diabetes or prediabetes, had a normal glycemic profile at the beginning of the hospitalization, and had average fasting blood glucose levels of 5.5 mmol/L (99 mg/dl) and a glycated hemoglobin (HbA1c) of 4.8% (29 mmol/mol) in October 2022. However, on December 16, 2022, she developed sudden and unexplained hyperglycemia at 24 mmol/L (432 mg/dl) without criteria for hyperosmolar hyperglycemic state or diabetic ketoacidosis (osmolarity at 296 mmol/kg H_2_O, pH 7.36, capillary ketone bodies at 0.1 mmol/L). Despite the initiation of a basal–bolus subcutaneous therapy with neutral protamine Hagedorn (NPH) and insulin aspart, her glycemia remained high. Normal glycemic profile could be reached with the introduction of continuous intravenous (IV) rapid insulin from December 18 to 19. From December 20, the treatment was switched to insulin glargine U100 and aspart with 45 U and >30 U, respectively. These doses were progressively increased, reaching 55 U of insulin glargine and 80 U of insulin aspart by January 3. Despite the high total subcutaneous insulin dose, the patient remained hyperglycemic with a maximum of 38 mmol/L (684 mg/dl), necessitating the reintroduction of continuous IV rapid-acting insulin for 24 h. Upon resuming subcutaneous insulin, a switch was made to long-acting insulin glargine U300 and rapid-acting insulin lispro, hypothesizing that resistance developed against the previous insulin analogs. A titration upwards to 75 U of long-acting insulin and around 100–110 U of rapid-acting insulin was needed, without preventing hyperglycemia up to 25 mmol/L (450 mg/dl). The IV rapid-acting insulin was again reinstated from January 31 for 72 h. Subsequently, the basal subcutaneous insulin dosage was increased to 110 U with variable doses of human insulin (Actrapid©; used in a subcutaneous route due to the same hypothesis previously mentioned) during the remainder of the hospitalization, ranging from 6 to 14 U, excluding corrections (**Figure 1**).

Treatment adjustments throughout the patient’s stay were complicated by rare episodes of hypoglycemia between episodes of hyperglycemia. Each hyperglycemic episode was accompanied by hyperosmolarity without criteria for hyperosmolar hyperglycemic state or ketoacidosis (**Table 1**). The patient exhibited chronic metabolic acidosis with a normal anion gap and hyperkalemia, attributed to type 4 renal tubular acidosis. No oral antidiabetic medications were prescribed due to hepatic and renal insufficiency.

**Table 1:** Biological factors, neoplastic factors, and autoantibodies.

	Date	Value	Reference range
Biological factors			
HbA1c	1.10.2022	4.8%	Normal: <5.7% Prediabetes: 5.7%–6.4% Diabetes: >6.4%
	20.12.2022	6.6%	
Ketone bodies	All the time	0.1–0.2	<0.3 mmol/L
C-peptide	29.12.2022	18.3	1–3.1 µg/L
	30.12.2022	20.1	
Autoantibodies in diabetes			
Anti-insulin	18.12.2022	0.19	<0.4 E/ml
	05.01.2023	0.23	
Anti-IA2	18.12.2022	<15	<0.15 U/ml
Anti-GAD	18.12.2022	<5	<10 IE/ml
Anti-ZnT8	18.12.2022	3.4	<15 U/ml
Anti-islet	18.12.2022	Negative	<1:10
Neoplastic markers			
IGF-1	06.01.2023	25.4	72.8–212 µg/L
Alpha-1-fetoprotein (AFP)	17.01.2023	1.3	<5 kUI/L

The patient received both enteral and parenteral nutrition support from January 27 to February 11 [body mass index (BMI) = 19.3 kg/m^2^, weight = 46 kg].

The patient had normal residual insulin secretion, indicating preserved pancreatic function (**Table 1**). The various antibody tests and workup for paraneoplastic syndrome were negative (**Table 1**), and the different imaging studies (CT scan of the brain, thorax, abdomen, and pelvis and lymphoscintigraphy of the lower limbs) were normal. Furthermore, a thorough clinical examination of the skin was performed to rule out lipodystrophies or other dermatological conditions, and multiple insulin injection sites were examined (arms, thighs, and abdomen).

Before the onset of hyperglycemia, adrenal insufficiency was suspected due to the persistence of hyponatremia and hyperkalemia. The cosyntropin stimulation test showed cortisol in the gray zone: T0 = 152 nmol/L, T60 = 433 nmol/L. Hydrocortisone 30 mg/day was initiated from December 10; however, due to the onset of hyperglycemia, the dosage was reduced to 15 mg/day on December 18 (it was not considered as a causal factor due to the relatively low doses).

The patient was finally transferred to the Geneva University Hospitals on February 12, 2023, for liver transplantation. Insulin therapy could then be tapered and successfully discontinued afterward with a normal/non-diabetic glycemic profile. After an immunosuppressive induction therapy with mycophenolate mofetil, tacrolimus, basiliximab, and methylprednisolone, the ongoing immunosuppressants were prednisone and tacrolimus. In the following month, the patient developed a post-transplantation diabetes mellitus, which was initially managed with insulin and currently treated solely with a GLP-1 receptor agonist.

## Discussion

Patients living with liver cirrhosis often show impaired glucose tolerance and hyperinsulinemia suggesting IR (1), with 17%–30% being clinically “diabetic” (5). This association, sometimes called hepatogenic diabetes (HD), is not recognized by the American Diabetes Association and the World Health Organization as a specific type of diabetes (6). HD is less associated with risk factors such as a family history of diabetes, BMI, and age than T2DM (2). The assessment, pathogenesis, therapeutic strategies, and death causes of hepatogenous IR vary from those of T2DM (3). Its exact mechanism remains unclear (7). Distinctive factors including hepatic parenchymal cell damage, portal systemic shunting, and the liver disease etiology are responsible for the development of hepatogenic IR (3, 4). IR occurs when normal levels of insulin do not produce a sufficient metabolic response and require higher amounts to achieve normal glycemic levels (8). During the first-pass metabolism of insulin, 60% secreted by the pancreas into the portal system is degraded by the liver (9). As blood glucose enters the liver via the portal vein, hyperinsulinemia could be explained either by portosystemic shunt or by hepatic parenchymal damage in patients with liver cirrhosis (3) and is enhanced by high concentrations of antagonistic hormones (e.g., insulin-like growth factor, growth hormone, and glucagon) (5). Nevertheless, diabetes is not always present in patients with cirrhosis. Environmental factors and the cause of the liver disease may play a role (2). Even in the absence of portosystemic shunt and hepatic parenchymal injury, patients with chronic hepatitis C virus infection may present hyperinsulinemia, possibly due to increased IR (5, 10). Peripheral tissue (muscular and adipose) IR also plays a key role in impaired glucose tolerance (5).

Pancreatic islet hypertrophy found on the biopsies of patients with cirrhosis suggests that increased IR may be an adaptive response of pancreatic beta cells leading to hyperinsulinemia (3).

There is also a presumption that the activity of pancreatic beta cells in insulin secretion could be altered by various factors (e.g., environment, genetics, and the etiology of the liver disease) (5). For example, in alcohol-related liver disease, the development of diabetes can occur from excessive alcohol consumption by reducing the insulin-mediated glucose uptake and damaging pancreatic islet cells (2). These mechanisms could not explain the IR in our patient as there was alcohol abstinence for months and the C-peptide values were normal.

In patients with cirrhosis, IR is known to accelerate the progression of liver disease (11). HD is less commonly associated with microangiopathy, and the main causes of death in these patients are related to their liver disease (3, 5). This suggests that a greater degree of liver failure and mortality is reflected in the development of diabetes in patients with cirrhosis (6, 12) and may even be associated with the development of hepatocellular carcinoma (13, 14). There is currently no prognostic score of cirrhosis that incorporates glucose intolerance or diabetes (5, 15).

In patients with HD, acceleration of liver fibrosis and inflammation leading to severe liver failure might worsen the evolution of cirrhosis, while a higher risk of bacterial infections can increase mortality (5, 16). Assessment of diabetes in cirrhotic patients is complex, and HbA1c is an imprecise marker for diabetes diagnosis and management [5] as the reference ranges assume normal erythrocyte lifetime. Falsely low HbA1c levels can result from any condition that reduces the survival of red blood cells, such as hemolysis due to hypersplenism or cirrhosis (6).

The management of HD is complex and depends on several parameters such as the nutritional status of the patient, the risk of hypoglycemia, and the degree of hepatic or renal insufficiency (5). For mildly hyperglycemic patients, one of the safe first-line treatments is diet and exercise; however, malnutrition affects many people with cirrhosis, and hypoalbuminuria may be exacerbated by dietary restriction, worsening the prognosis (6). Concerning oral antidiabetic medications, most of them are metabolized in the liver, and some carry a high risk of hepatotoxicity or lactic acidosis in cirrhotic patients (3). Insulin, on the other hand, requires close follow-up (5).

In our patient, the need for abnormally high doses of subcutaneous insulin remains unclear. This could be explained by a rare syndrome known as subcutaneous IR, defined by severe resistance to subcutaneous insulin with normal response to IV insulin therapy (17–20). The underlying pathophysiology is thought to involve the increased degradation or sequestration of subcutaneous insulin, resulting in impaired absorption (18, 21). However, as illustrated in **Figure 1**, while the patient’s need for IV insulin was comparatively lower than that for subcutaneous insulin, it remained significantly elevated. The patient required more than 1 IU kg^−1^ day^−1^ of IV insulin to achieve adequate glycemic control despite the advantages of IV insulin, which include rapid action and more precise dose adjustments. In classic cases of subcutaneous IR, the response to IV insulin is typically much more favorable, with substantially lower insulin requirements. This suggests that the mechanisms of our patient’s condition are beyond subcutaneous insulin degradation or sequestration.

Finally, liver transplantation, as seen in our patient, can reverse diabetes in the majority of patients by restoring normal insulin sensitivity (22). Improved clearance of glucose by the liver and its disposal in the peripheral circulation are thought to be responsible for this effect (5).

The uniqueness and the rarity of this case make it a valuable contribution to the literature. However, the absence of access to the patient’s blood and urine samples prevented us from conducting additional laboratory investigations that could have provided further insights into and understanding of the mechanisms of the HD in our patient.

## Conclusion

Our case report highlights the reversibility of diabetes secondary to liver transplantation, likely through the recovery of insulin sensitivity (including insulin receptors) and glucose disposal. Further standardized and controlled studies are needed to clarify the pathophysiological mechanisms underlying IR in patients with cirrhosis, given the major differences in the clinical presentation and behaviors of HD compared with T2DM. As reported in our case, the association with resistance to subcutaneous insulin syndrome can add to the difficulties of management, and liver transplantation appears to be a curative response in some cases.

## Data Availability

The original contributions presented in the study are included in the article/Supplementary Material. Further inquiries can be directed to the corresponding authors.
